# A Clinicopathological and Immunohistochemical Correlation in Cutaneous Metastases from Internal Malignancies: A Five-Year Study

**DOI:** 10.1155/2014/793937

**Published:** 2014-08-25

**Authors:** Sarita Nibhoria, Kanwardeep Kaur Tiwana, Manmeet Kaur, Sumir Kumar

**Affiliations:** ^1^Department of Pathology, G.G.S. Medical College & Hospital BFUHS, Faridkot, Punjab 151203, India; ^2^Department of Skin and VD, G.G.S. Medical College & Hospital BFUHS, Faridkot, Punjab 151203, India

## Abstract

Cutaneous metastases from internal malignancies are uncommon and occur in 0.6%–10.4% of all patients with cancer. In most cases, cutaneous metastases develop after the initial diagnosis of the primary internal malignancy and late in the course of the disease. Skin tumors are infrequent in Asian population and cutaneous metastases are quite rare. Cutaneous metastases carry a poor prognosis with average survival of few months. In the present five-year study 1924 malignant tumors were screened which included only nine cases of cutaneous metastatic deposits. A wide range of site and clinical presentations including nodules, plaques, and ulcers was noted. Histopathological findings were significant and corresponded with the primary internal malignancy. Cutaneous metastases from breast carcinoma (44.4%) were the most common finding followed by non-Hodgkin lymphoma and renal cell carcinoma (22.2% each) and carcinoma cervix (11.1%). The aim of our study is to classify the cutaneous metastases and to evaluate their clinicopathologic and immunohistochemical correlation with the primary tumor.

## 1. Introduction

Cutaneous metastasis can be defined as the spread of a tumor from the site of its primary origin to the skin [1]. Skin metastasis may be the first sign of an advanced cancer or an indicator of cancer recurrence [2, 3]. Up to 9% of patients with cancer may develop skin metastases, while metastasis may develop more than 10 years after initial cancer diagnosis [3]. A wide morphologic spectrum of clinical appearances has been described in cutaneous metastases including nodules, plaques, papules, tumors, and ulcers [4]. While carcinomas are the most common type of cancer to metastasize, sarcomas, lymphomas, and leukemias also represent a substantial percentage of all skin metastases [5]. The relative frequencies of metastatic skin disease in each sex correlate with the frequency of different types of primary cancer. Thus women with the skin metastases have the following distribution in decreasing order of primary malignancies: breast, ovary, oral cavity, lung, and large intestine. In men, the distribution is as follows: lung, large intestine, oral cavity, kidney, breast, esophagus, pancreas, stomach, and liver [4]. Generally, cutaneous metastases herald a poor prognosis with average survival time of a few months.

## 2. Materials and Method

In the present five-year study, patients diagnosed with an internal malignancy including hematolymphoid neoplasms, registered between March 2009 and March 2014 in the Pathology Department, were consecutively screened. The H&E stained histopathological sections of skin biopsies received in the Pathology Department were reevaluated. The inclusion criteria were cases of cutaneous metastatic deposits with or without known primary malignant tumor. Cases with direct extension of primary malignancy into the overlying skin were excluded. Physical and dermatologic examination details were obtained from the patient files and histopathology requisition forms. The clinical presentation, site, and histopathological details, especially those suggesting the primary tumor site, were evaluated along with the secondary morphological changes in the skin tissue. Immunohistochemistry was performed on all except for one case of cutaneous metastases and correlation with the primary internal malignancy was done.

## 3. Results

In the present five-year study, a total of 1924 malignant tumors were screened which included nine cases of cutaneous metastatic deposits. The cutaneous metastases were seen more in females (5 out of 9 patients). The four male patients had skin metastases from renal cell carcinoma and from non-Hodgkin lymphoma (2 cases each). The age range was found to be 30–72 with mean age 60 years.

A wide range of clinical presentations and regional localizations was noted. Plaque and nodule were the most frequent clinical presentation (4 cases each out of 9) followed by ulcer (1 case out of 9). The size of the skin lesions varied from 0.25 cm to 5.0 cm.

The regional localization in cases of breast carcinoma included chest (2 cases), chest and abdomen (1 case), and face, scalp, and trunk (1 case). Both cases of renal cell carcinoma showed deposits on abdomen. A case of non-Hodgkin lymphoma showed widespread skin deposits on face, scalp, and trunk while another case showed localized deposits on abdomen only. A single case of carcinoma cervix showed skin deposits on thigh (Table 1).

The duration of time after which the cutaneous metastases developed was variable and ranged from 10 months to five years. In majority of the cases, patients had prior history of a primary internal malignancy.

**Figure 1:**
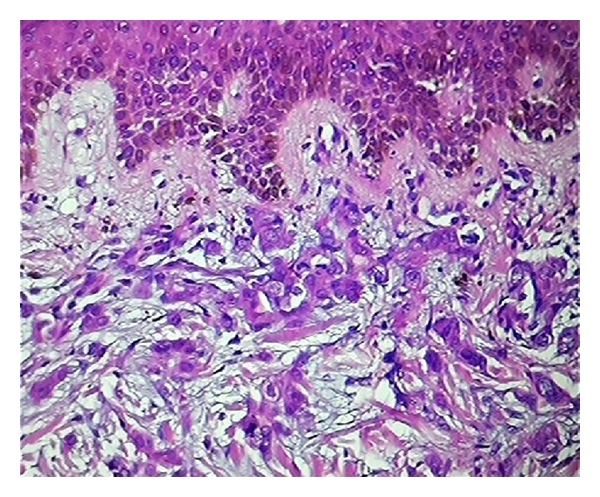
Sections show deposits of metastatic breast carcinoma (H&E ×100).

**Figure 2:**
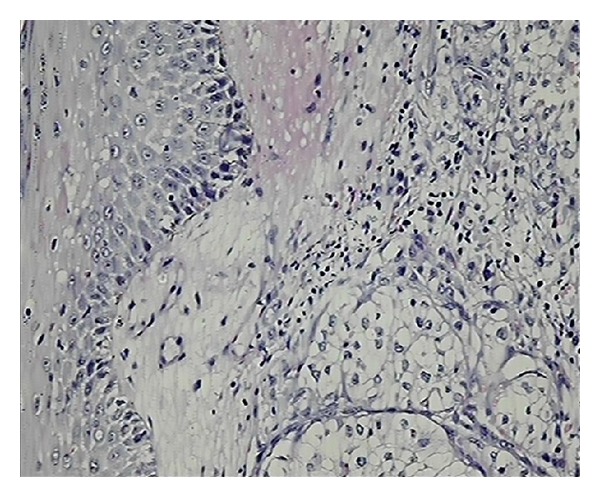
Sections show islands of clear tumour cells in dermis suggesting metastatic renal cell carcinoma (H&E ×100).

**Figure 3:**
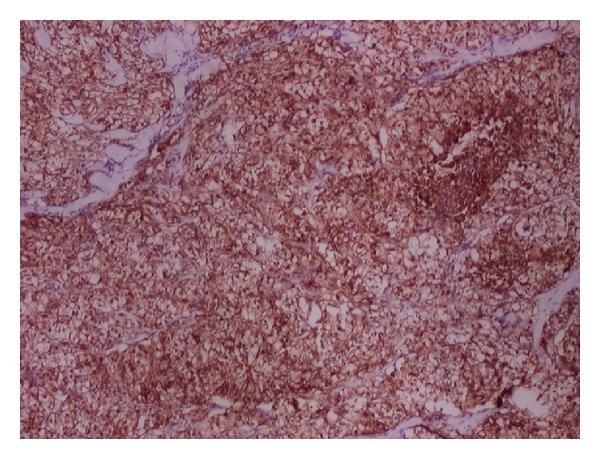
On IHC showing diffuse CD10 positivity confirming metastatic renal cell carcinoma (×40).

The histopathologic examination revealed significant findings. The morphological patterns and microscopic appearances suggested the likely tissue of origin. In cases of cutaneous metastases from carcinoma breast, the histologic examination revealed invasion of dermis and subcutis by groups, cords, and nests of tumor cells. The tumor cells were large with large pleomorphic nuclei. Fibrosis was evident in one case only (Figure 1). ER/PR positivity was seen in 3 out of 4 cases of cutaneous metastases from carcinoma breast. The metastatic deposits which were negative for ER/PR had previous reports of ER/PR negativity in the primary tumor too. The deposits of renal cell carcinoma showed presence of tumor cells in glandular configuration or in nests. The tumor cells showed oval nuclei with abundant, clear cytoplasm. IHC (CD-10) was applied and confirmed the diagnosis in both cases (Figures 2 and 3).

**Figure 4:**
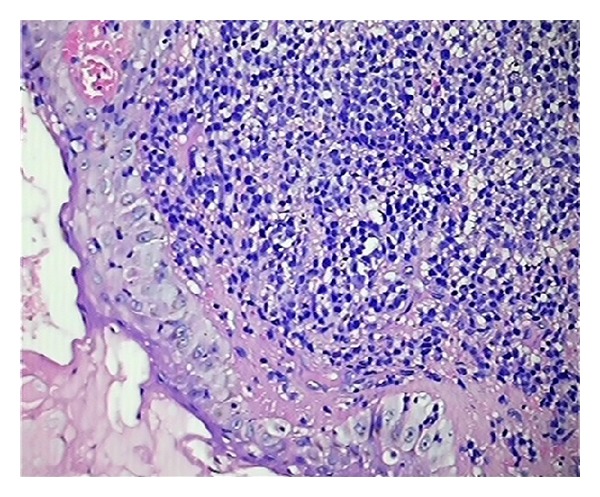
Sections show monomorphic sheets of non-Hodgkin lymphoma cells with spared epidermis (H&E ×100).

**Figure 5:**
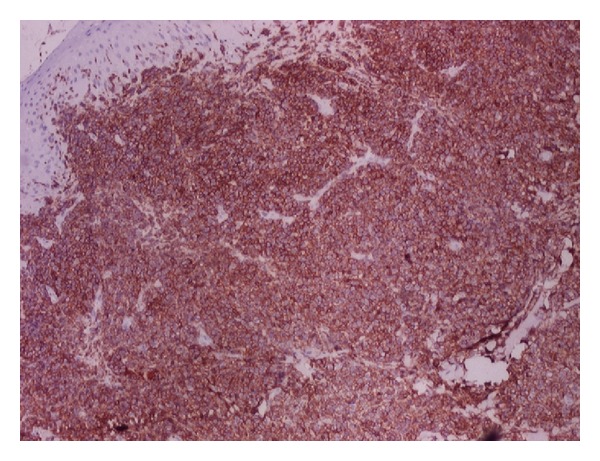
On IHC showing diffuse CD45 positivity confirming metastatic non-Hodgkin lymphoma (×40).

The deposits of non-Hodgkin lymphoma showed diffuse presence of atypical lymphoid cells in the dermis and subcutis. The atypical lymphoid cells showed finely stippled chromatin, inconspicuous nucleoli, and sparse cytoplasm. There was no evidence of epidermotropism which ruled out the possibility of primary cutaneous lymphoma. Leucocyte common antigen (CD 45) was positive in both of the cases (Figures 4 and 5).

The deposits of carcinoma cervix showed presence of tumor cells arranged in groups and nests. The tumor cells were large and showed vesicular nuclei and moderate amount of eosinophilic cytoplasm. As the epidermis was not involved, the differentiation of cutaneous metastatic deposits from primary squamous cell carcinoma was possible. Moreover, a prior history of squamous cell carcinoma of cervix three years back also suggested metastatic skin deposits.

In all the cases of cutaneous metastatic deposits, the main challenge for the pathologist is to exclude the possibility of primary skin neoplasms (including benign and malignant adnexal tumors) and inflammatory conditions of skin especially in cases of skin deposits of NHL. Immunohistochemistry supported the histologic diagnosis and correlated well with the primary internal malignancy.

## 4. Discussion

Cutaneous metastases occur infrequently and are rarely present at the time the cancer is initially diagnosed [6]. Cutaneous metastases occur in 0.6%–10.4% of all patients with cancer and represent 2% of all skin tumors [4]. In the present five-year study, only 9 cases out of a total of 1924 patients with internal malignancies presented with cutaneous metastases, thus showing a prevalence rate of approximately 0.5% which is near to the lower limit of the reported range.

Cutaneous metastases may either be the initial manifestation of an internal malignancy or represent recurrent neoplastic disease [7]. In the present study, all the nine patients had prior history of primary internal malignancy, thus representing the recurrence of the primary tumor. A landmark study by Brownstein and Helwig from 1972 found the most common tumors to metastasizing to the skin were breast, lung, colorectal, and melanoma [8]. While Gul et al. in their study found that most common cancer types metastasizing to skin were breast, colon, and ovary in females and lung and colon cancers in males. In the present study, the order of cutaneous metastases from internal malignancy was carcinoma breast (4/9 cases) and carcinoma cervix (1/9 case) in females while it was renal cell carcinoma and non-Hodgkin lymphoma (2/9 cases each) in males. Cutaneous metastases from carcinoma breast were the most common finding which is in concordance with the above mentioned studies.

According to Basu and Mukherjee, gynecological malignancies rarely give rise to metastatic deposits on the skin [9]. Skin metastasis from uterine cervical carcinoma is a rare event with the reported incidence ranging from 0.1 to 2% [10]. Our study also included a single case of cutaneous metastasis from carcinoma cervix which is quite rare.

Skin metastases of renal cell carcinoma are not easily identified because of the low suspicion index for these skin lesions, which usually mimic common dermatologic disorders. Skin metastases of renal cell carcinoma have been reported to occur in around 3% of renal tumors and are more common in males [11]. In our study we reported two cases of cutaneous metastases from renal cell carcinoma and both the patients were males.

Cutaneous metastases were most frequently (2.6%) seen in cases with hematological malignancies in a study done by Gul et al. [7], while in the present study cutaneous metastases from NHL and RCC were the most common tumors in males.

It has been observed that many carcinomas spread through the lymphatic route to areas having common lymphatic drainage as that of the primary site [12]. In the present study as well, the skin deposits from breast carcinoma were mainly localized to the chest wall. Other cutaneous deposits have also shown corresponding patterns of skin localization. Scalp is relatively a rare site for the localization of skin metastases [8]. In our study, two out of nine cases showed cutaneous metastases on scalp. According to Lookingbill et al., the most common presentation of cutaneous metastatic deposits is multiple nodular lesions [6]. Contrary to that nodules and plaque were the most common clinical presentation in our study.

Metastatic carcinomas are usually differentiated from primary skin carcinomas because of the latter's typical histological patterns, the epidermal connection, intraepidermal/intra-adnexal (in situ component) tumor, or the presence of a benign counterpart [3, 6]. In cases where distinction between metastatic and primary skin tumor is difficult, a variety of immunohistochemical staining panels can be helpful [13–17]. In our study too, IHC applied in eight out of nine cases correlated well with the primary tumor. Tumor markers are becoming increasingly important in breast cancer research because of their impact on prognosis, treatment, and survival [18]. The ER and PR markers used in skin deposits from breast carcinoma confirmed the primary tumor. According Bauer et al., ER, PR, and HER-2 neu negative breast cancers affect younger women and were more aggressive and these women had poorer survival regardless of stage [18]. In two cases, histopathological diagnosis was clear cell carcinoma which on immunostaining with CD 10 was confirmed as metastatic renal cell carcinoma. Similarly, LCA positivity confirmed the metastatic deposits of NHL in other two cases.

To conclude, cutaneous metastases occur infrequently and that internal malignancy rarely presents with skin involvement. However, early diagnosis is necessary which may have profound effect on patient management and survival. Immunohistochemistry is an important ancillary aid in the diagnosis of cutaneous metastases.

## Figures and Tables

**Table 1:** Table depicting the summary of the study.

Sample number	Age (years)	Sex	Primary internal malignancy	Site of cutaneous metastasis	Clinical presentation	Duration of appearance of cutaneous metastasis
Case number 1	65 years	Female	Carcinoma cervix	Left thigh	Nodules	3 years
Case number 2	72 years	Male	Renal cell carcinoma	Abdomen	Nodules	5 years
Case number 3	70 years	Male	Non-Hodgkin lymphoma	Abdomen	Plaque	1 year
Case number 4	55 years	Female	Carcinoma breast	Chest	Plaque	2 years
Case number 5	50 years	Female	Carcinoma breast	Chest	Ulcers	3 years
Case number 6	30 years	Male	Non-Hodgkin lymphoma	Scalp, face, trunk	Plaques	10-11 months
Case number 7	60 years	Female	Carcinoma breast	Scalp, face, trunk	Nodules	3 years
Case number 8	72 years	Male	Renal cell carcinoma	Abdomen	Plaque	3 years
Case number 9	66 years	Female	Carcinoma breast	Chest, abdomen	Nodules	2.5 years
